# A novel immune-related gene signature for predicting immunotherapy outcomes and survival in clear cell renal cell carcinoma

**DOI:** 10.1038/s41598-023-45966-8

**Published:** 2023-11-02

**Authors:** Jie Gu, Xiaobo Zhang, ZhangZhe Peng, Zhuoming Peng, Zhouning Liao

**Affiliations:** 1 Department of Geriatric Urology, Xiangya International Medical Center, Xiangya Hospital, Central South University, Hunan Province, Changsha, 410008 China; 2 National Clinical Research Center for Geriatric Disorders, Xiangya Hospital, Central South University, Changsha, 410008 Hunan Province China; 3 Department of Nephrology, Xiangya Hospital, Central South University, Changsha, 410008 Hunan Province China; 4 Department of Respiratory and Intensive Care Medicine, Union Shenzhen Hospital, Huazhong University of Science and Technology, Shenzhen, 518000 Guangdong Province China

**Keywords:** Immunology, Biomarkers, Medical research, Molecular medicine, Nephrology, Pathogenesis

## Abstract

Clear cell renal carcinoma (ccRCC) is one of the most common cancers worldwide. In this study, a new model of immune-related genes was developed to predict the overall survival and immunotherapy efficacy in patients with ccRCC. Immune-related genes were obtained from the ImmPort database. Clinical data and transcriptomics of ccRCC samples were downloaded from GSE29609 and The Cancer Genome Atlas. An immune-related gene-based prognostic model (IRGPM) was developed using the least absolute shrinkage and selection operator regression algorithm and multivariate Cox regression. The reliability of the developed models was evaluated by Kaplan–Meier survival curves and time-dependent receiver operating characteristic curves. Furthermore, we constructed a nomogram based on the IRGPM and multiple clinicopathological factors, along with a calibration curve to examine the predictive power of the nomogram. Overall, this study investigated the association of IRGPM with immunotherapeutic efficacy, immune checkpoints, and immune cell infiltration. Eleven IRGs based on 528 ccRCC samples significantly associated with survival were used to construct the IRGPM. Remarkably, the IRGPM, which consists of 11 hub genes (SAA1, IL4, PLAUR, PLXNB3, ANGPTL3, AMH, KLRC2, NR3C2, KL, CSF2, and SEMA3G), was found to predict the survival of ccRCC patients accurately. The calibration curve revealed that the nomogram developed with the IRGPM showed high predictive performance for the survival probability of ccRCC patients. Moreover, the IRGPM subgroups showed different levels of immune checkpoints and immune cell infiltration in patients with ccRCC. IRGPM might be a promising biomarker of immunotherapeutic responses in patients with ccRCC. Overall, the established IRGPM was valuable for predicting survival, reflecting the immunotherapy response and immune microenvironment in patients with ccRCC.

## Introduction

Renal cell carcinoma (RCC) is one of the most prevalent cancers worldwide, accounting for nearly 90% of all kidney cancers^1,2^. Its incidence is approximately twice as high in men as in women^3^. RCC consists of three main histological subtypes with different molecular and genetic characteristics; clear cell renal cell carcinoma (ccRCC) is the most extensive histological subtype (approximately 80%)^4^. Even in patients with localized ccRCC, surgical resection, which is considered the best treatment choice, is associated with a high rate of recurrence^5,6^. However, due to the anatomical location of the kidneys early symptoms in many patients with ccRCC are subtle and easily overlooked leading to delayed diagnosis. Consequently, by the time of diagnosis, metastases are already advanced in many cases^7,8^. Over the past 20 years, there has been limited progress in ccRCC prognosis. It is essential to investigate the mechanisms underlying ccRCC in detail and develop novel therapeutic targets to enhance the survival of patients with ccRCC^9,10^.

The immune system is closely linked to the prognosis of malignant tumors^11^. Immuno-oncology, the immune microenvironment, and immune cells are essential factors in tumorigenesis^12,13^. All cancers can be considered immune to some extent, and immunotherapy exploits the host immune system to produce a specific immune response to identify and eliminate cancer cells, thereby reducing the incidence of tumor metastasis and recurrence^14,15^. The tumor immune microenvironment (TIME) is integral to immunotherapy and has gradually gained increasing attention. Analysis of the TIME can help improve responsiveness to immunotherapy. Additionally, TIME can be used as a significant prognostic indicator to enhance the efficacy of precise treatments^16,17^. Considering the vital importance of the TIME, the immediate task is to build a signature of immune-related genes (IRGs) that are closely related to the TIME to explore the IRGs of patients with ccRCC.

Although ccRCC signatures based on IRGs have been identified recently, a more comprehensive and credible indicator is urgently needed. It is possible to predict both survival and immunotherapy in ccRCC patients^18,19^. Therefore, in this study, an IRG-based prognostic model (IRGPM) was established through cancer bioinformatics and genomics, and its reliability was validated using several datasets. Moreover, the value of IRGPM in the survival of patients with ccRCC, together with its potential predictive role in immunotherapy, was examined. Finally, the IRGPM can guide clinicians on cancer immunotherapy in patients with ccRCC.

## Methods

### Acquisition of sample information

Clinical information together with RNA-seq data for the samples of ccRCC were gathered from The Cancer Genome Atlas (TCGA) (https://portal.gdc.cancer.gov/) and Gene Expression Omnibus (GEO) (https://www.ncbi.nlm. nih.gov/gds/) and named for the TCGA-KIRC (n = 528) and GSE29609 (n = 39). TCGA-KIRC and GSE29609 are classified as training cohort and validation cohort, separately. Furthermore, 1793 IRGs were acquired from The Immunology Database and Analysis Portal (ImmPort) database (https://www.immport.org/home)^20^.

### Construction of differentially expressed IRGs (DEIRGs)

Differentially expressed genes (DEGs) between ccRCC and normal samples were identified using the R package “edgeR” and “DESeq2” based on the following criterion: false discovery rate (FDR) < 0.05 and |log2 fold-change (FC)|> 1.5^21,22^. In addition, DEIRGs were obtained by analysis of the crossover of IRGs and DEGs. The R package “ggplot2” was employed to visualize the DEIRGs using the Venn diagram and create a volcano plot of DEIRGs^23^.

### Biological function analysis

To study the bio-functions of DEIRGs, we conducted functional enrichment analyses of DEIRGs, including the Kyoto Encyclopedia of Genes and Genomes (KEGG) and Gene Ontology (GO), with the R package “clusterProfiler”^24^. The GO terms cover biological process (BP), molecular function (MF) and cellular constituents (CC)^25–28^. The signaling pathways of KEGG and GO terms were enriched as measured by FDR < 0.01. This was followed by a visual analysis of the first 10 most critical KEGG signaling pathways and 15 most related GO terms applying the R package “ggplot2”^29^. For further investigation of the underlying mechanism of DEIRGs, Gene Set Enrichment Analysis (GSEA) was implemented to elucidate the significant biological process with the R package “clusterProfiler”^30^. P adjust < 0.05 and FDR < 0.25 were regarded as statistically significant.

### Signature exploitation and reliability assessment

IRGs related to prognosis were determined, and IRGPM based on the training set was built and then its predictive performance was validated in the GSE29609 cohort. Specifically, a univariate Cox proportional hazard regression analysis was implemented while investigating prognosis-related IRGs to assess the relationship between DEIRGs and overall survival (OS) in training set^31^. The prognosis-related IRGs were identified using the significance value of *P* ≤ 0.05. Thereafter, Least Absolute Shrinkage and Selection Operator (LASSO) was employed to selected the most relavant IRGs associated with prognosis via the R package “glmnet” and “survival”^32^. Lambda. min was chosen as the cutoff. Additionally, the IRGPM, which predicts the prognosis OS of ccRCC patients, was built applying Multivariate Cox proportional hazard regression analysis. In multivariate Cox regression analysis, the risk score of each patient with ccRCC was weighted based on their estimated regression coefficient. Patients were then classified as low- and high-risk groups using the median risk score as a threshold. Kaplan–Meier (K–M) analysis was performed for the comparison of survival between both groups via the R package “survival” to further validate the IRGPM's predictive power. Furthermore, the “time ROC” R package was applied for generating the ROC curve, which illustrates the sensitivity and specificity of IRGPM including 1-, 3-, and 5-year survival^33,34^.

### Relationship between IRGPM and clinical pathological factors

Univariate and multivariate Analysis of OS for clinical-pathological parameters together with IRGPM were implemented in TCGA-KIRC cohort, and GSE29609 through R package “survival”^35^. In addition, the correlation of IRGPM with different clinic-pathological parameters (sex, histologic state, M stage, N stage, pathologic stage, T stage) was assessed with Mann–Whitney U tests.

### Construction of prognostic nomogram

From the IGRPM and the clinical parameters (N-stage, M-stage, T-stage, pathological stage, Histological grade, and Sex), a nomogram was further built that could predict the probability of survival of patients suffering from ccRCC. Furthermore, calibration curves were drawn to check the predictive validity of nomograms, and the agreement between actual and predicted survival performance can also be compared. Nomograms along with its calibration curves were plotted with the R package “rms”.

### Evaluation of immune cell infiltration and checkpoint

R package “GSVA” was exploited to examine the immune cell infiltration between low- and high-riskscore groups^36,37^. The association between the immune checkpoints/immune cells and the model's risk score was assessed through the Mann–Whitney U test, including programmed cell death protein 1 (PD1/PDCD1), cytotoxic T-lymphocyte-associated protein 4 (CTLA4), PDCD1LG2, PD ligand 1 (PDL1/CD274), T cell immunoglobulin and ITIM domain (TIGIT), Sialic Acid Binding Ig Like Lectin 15 (SIGLEC15), lymphocyte activation gene-3 (LAG3) and Hepatitis A virus cellular receptor 2 (HAVCR2).

### Effectiveness of immunotherapy

Immunophenoscore (IPS) was employed to predict the immune checkpoint blockades (ICBs) responses in the TCGA-KIRC patients. IPS is calculated based on gene expression of four fundamental components: immunomodulators, MHC molecules, suppressor cells and effector cells, on scale ranging from 0 to 10. ccRCC patients’ IPS was obtained from The Cancer Immunome Atlas (TCIA)^38^.

### Statistical analysis

Statistical analyses were conducted in R software (version 3.6.3), with the Kruskal–Wallis test and Wilcoxon test adopted for non-normally distributed data between multiple and two groups, separately. While the one-way analysis of variance (ANOVA) and unpaired Student’s t-test were separately applied for the normally distributed variables between multiple and two groups. The R package “survminer” was exploited for the Kaplan–Meier survival plots. *P* < 0.05 illustrated a statistical significance, all statistical *P*-values were two-sided.

## Ethical approval and consent to participate

All data in this study were collected from public data- bases: TCGA and GEO. This article does not contain any studies with patients or animals performed by any of the authors.

## Results

### Analysis of DEIRGs

**Figure 1:**
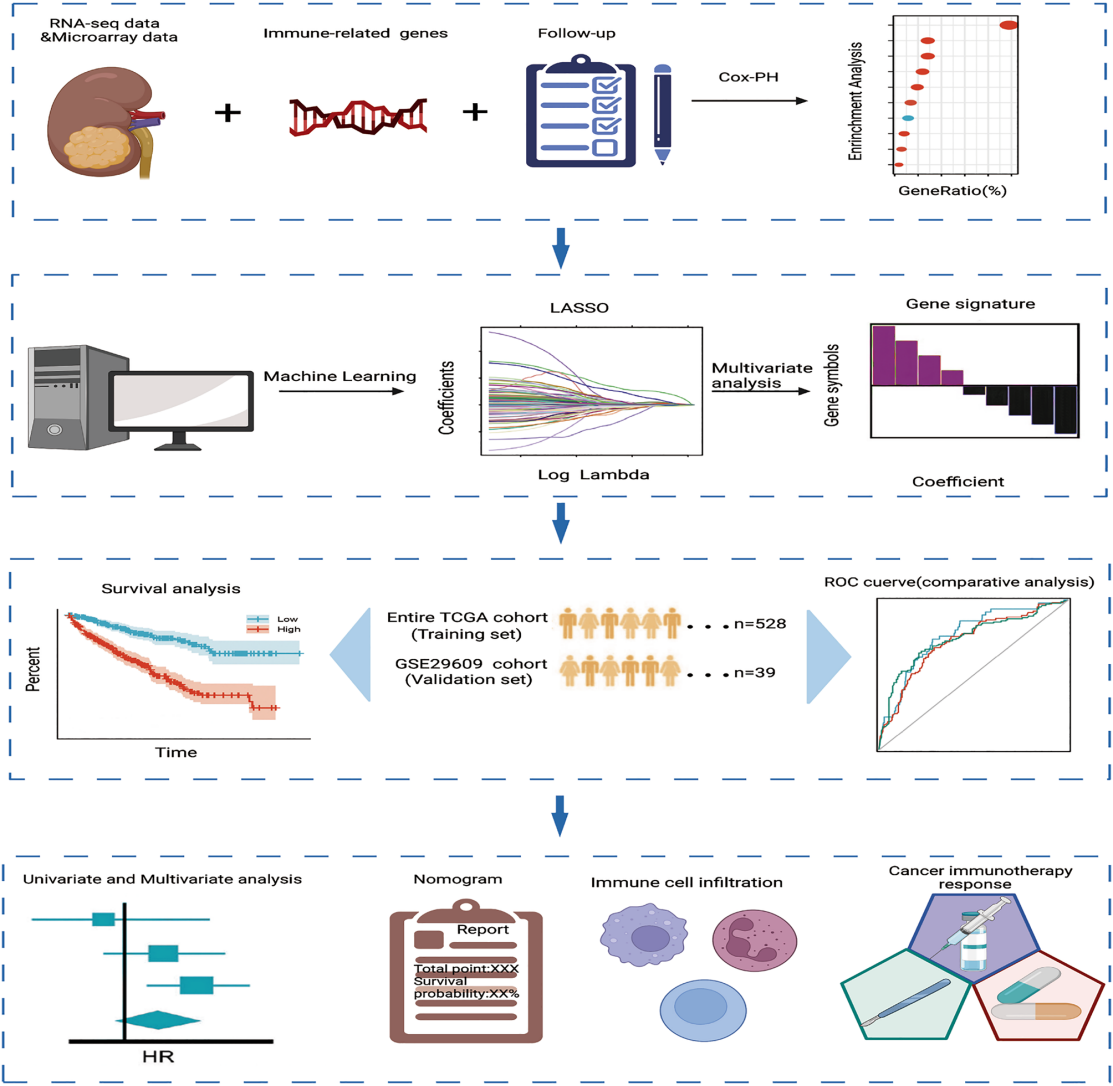
Experimental technical roadmap of the study.

**Figure 2:**
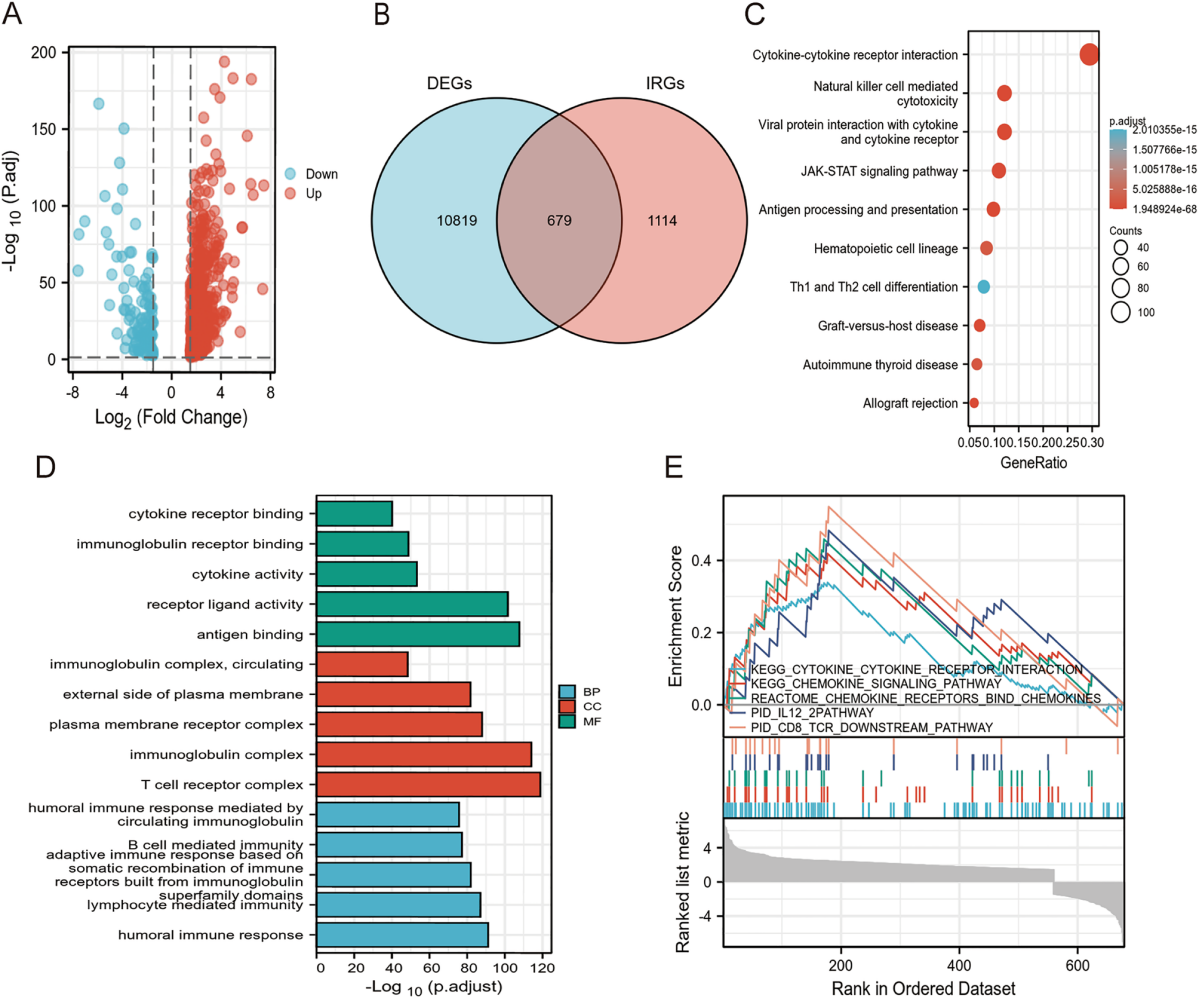
Identification of IRGPM. (**A**)Volcano plot for differentially expressed genes between normal patients and ccRCC based on TCGA-KIRC cohort. (**B**) Venn diagram of DEGs and IRGs. DEGs: Differentially expressed genes, IRGs: immune-related genes. (**C**) KEGG Analysis of the top 10 enrichment pathways. (**D**) GO enrichment analysis of 679 DEIRGs in ccRCC. BP, CC, and MF indicates molecular function, cellular constituents, and biological process, respectively. DEIRGs indicates differentially expressed immune-related genes. (**E**) GSEA revealed several significantly enriched oncological signatures. The upper part of the plot shows the enrichment score; the lower part of the plot shows the ranked list metric for the gene set.

Figure 1 provides a comprehensive flowchart of the study. Analysis of 528 ccRCC samples and 72 normal samples identified 11,498 DEGs. Meanwhile, 679 DEIRGs were obtained from the crossover of 11,498 DEGs and 1793 IRGs, including 559 increased and 120 decreased genes (Fig. 2A,B and Table S1). Functional enrichment analysis revealed that the top three relevant signaling pathways for DEIRGs were cytokines-cytokine receptor interaction, viral protein interaction with cytokines and cytokine receptors, and natural killer cell-mediated cytotoxity. (Fig. 2C and Table S2). Besides, the most enriched terms of BP, CC, and MF are “humoral immune response”, “T cell receptor complex” and “antigen binding”, respectively (Fig. 2D and Table S3). GSEA analysis has indicated strong associations between DEIRGs and oncological pathways such as response chemokine receptors binding chemokines, chemokine signaling pathways and cytokine-cytokine receptor interaction (Fig. 2E and Table S4).

### Construction of IRGPM

Univariate -Cox regression analysis was carried out for screening the OS-associated DEIRGs of KIRC patients and then 193 prognostic DEIRGs were determined (*P* < 0.05, Table S5). Subsequently, LASSO Cox analysis was conducted based on the 193 prognostic DEIRGs, and lambda.min was chosen to prevent overfitting during the procedure (Fig. S1). In total, 11 hub genes were converged after tenfold cross-validation, which were *SAA1*, *IL4, PLAUR, PLXNB3, ANGPTL3, AMH, KLRC2, NR3C2, KL, CSF2,* and *SEMA3G*. Then the multivariate Cox analysis was conducted for the establishment of IRGPM. Finally, 11 OS-related DEIRGs were identified and applied to establish a multivariate Cox IRGPM (Fig. S2). The IRGPM’s risk score was calculated via their coefficients of hub genes in the following way: Risk score = (Expression level of SAA1 × 0.037455) + (Expression level of IL4 × 0.493798) + (Expression level of PLAUR × 0.129711) + (Expression level of PLXNB3 × 0.232950) – (Expression level of ANGPTL3 × 0.117101) + (Expression level of AMH × 0.163395) + (Expression level of KLRC2 × 0.212960) – (Expression level of NR3C2 × 0.210899) – (Expression level of KL × 0.666003) + (Expression level of CSF2 × 0.297717) – (Expression level of SEMA3G × 0.408205).

### IRGPM predicts survival of ccRCC patients

**Figure 3:**
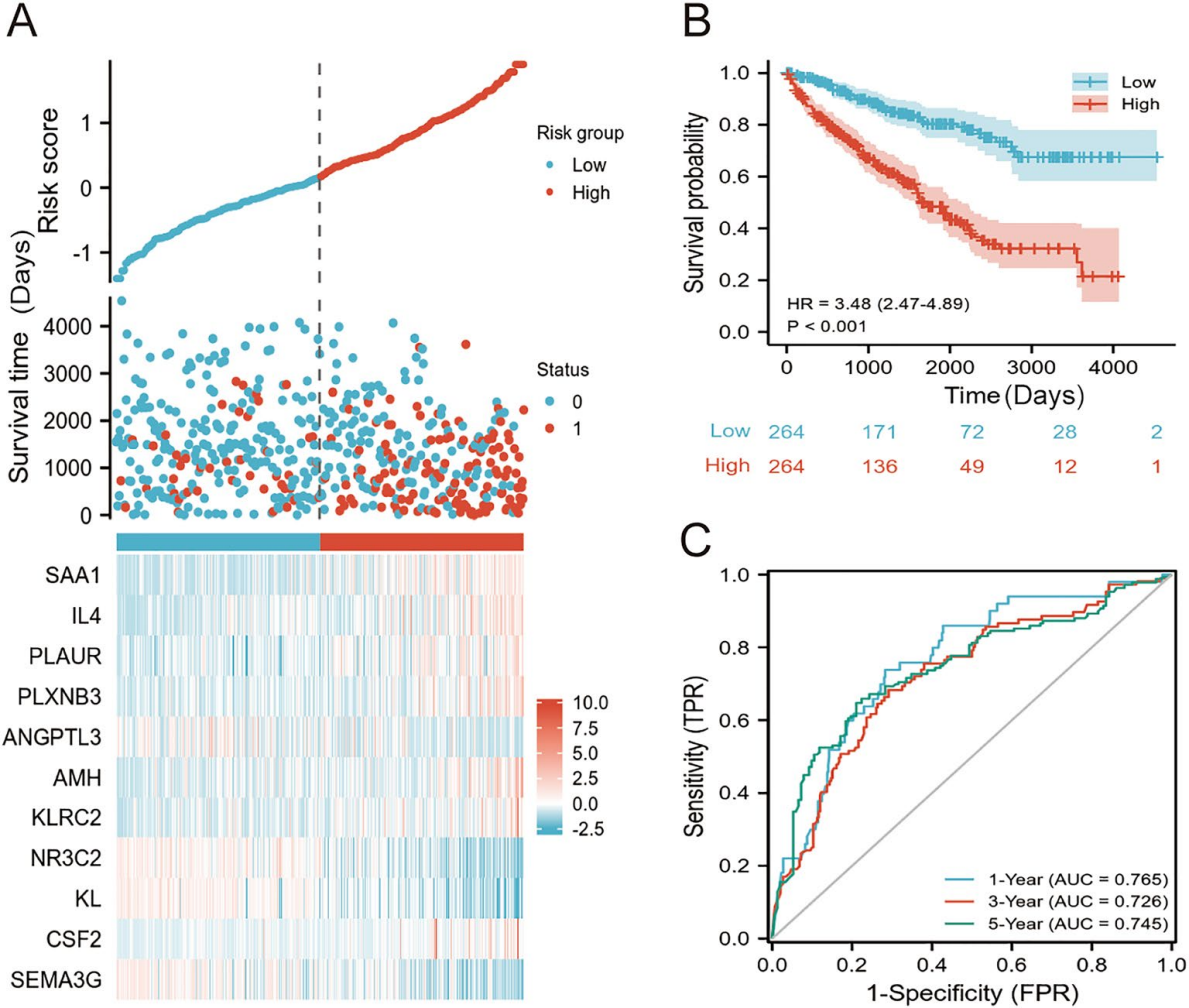
IRGPM precisely predicts the survival status of ccRCC patients. (**A**) Distribution of risk scores, expression of 11 OS-related DEIRGs, and survival status for ccRCC patients in low- and high-risk groups based on TCGA-KIRC cohort. “0” indicates alive and “1” indicates dead (**B**) The Kaplan–Meier curves for overall survival between low- and high-risk groups based on TCGA-KIRC cohort. (**C**) Analysis of the IRGPM's time-dependent ROC curve for 1-, 3-, and 5- years of survival based on TCGA-KIRC cohort.

Patients suffering from ccRCC were classified as high- and low-riskscore groups according to median IRGPM risk score (Fig. 3A and Table S6). In comparison with patients in the low riskscore group, those in the high riskscore group present a marked lower OS (Fig. 3B). The IRGPM was validated in the GSE29609 cohort, which also showed a high performance in predicting the OS (Fig. S3). Moreover, the time-dependent ROC curve illustrates the reliability of IRGPM (Fig. 3C). In the TCGA-KIRC cohort, for 1, 3 and 5-year survival, the area under the curve (AUC) was 0.765, 0.726 and 0.745, respectively, it shows that the constructed IRGPM is useful in monitoring the survival rate.

### IRGPM is significantly associated with disease progression

**Figure 4:**
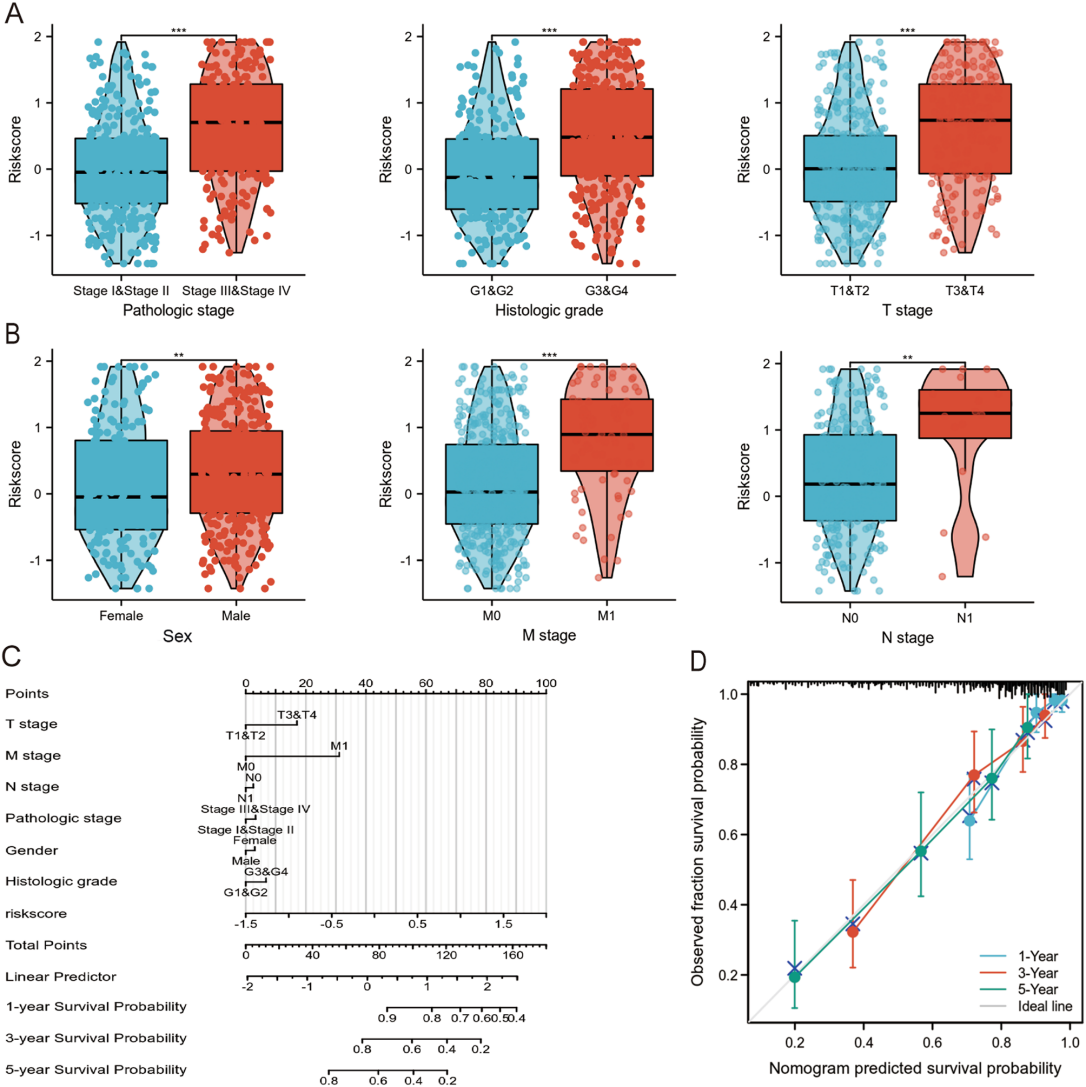
IRGPM risk score with different clinicopathological factors in ccRCC patients. (**A** and **B**) The association between IRGPM and clinicopathological factors in the TCGA-KIRC cohort. (**C**) Nomogram model predicting 1-, 3- and 5-year OS for ccRCC patients in TCGA-KIRC cohort. (**D**) The 1-, 3- and 5-year calibration curve of the nomogram in TCGA-KIRC cohort. The ideal line at 45° indicates a perfect prediction, the blue, red, green lines respectively represent 1‐, 3‐, and 5-year survival.

Analysis of the correlation between IRGPM and multiple clinicopathological parameters was investigated by the Mann–Whitney U test. In the TCGA-KIRC cohort, the high risk score group is positively related to patients with advanced pathologic stage, advanced histologic grade, advanced TNM stage, and male sex (Fig. 4A,B). The findings indicate that IRGPM is associated significantly with diverse clinicopathological factors, with higher riskscore corresponding to poorer clinicopathological status.

Moreover, we built a prognostic nomogram according to IRGPM and important clinicopathological parameters, to give a predictable quantitative analysis tool for predicting the patients' survival risk (Fig. 4C). Furthermore, the calibration curves of prognostic nomograms indicated excellent agreement between predicted values and the actual 1, 3 and 5-year survival rates in the TCGA-KIRC cohort (Fig. 4D).

### IRGPM predicts the immune cell infiltration of the ccRCC microenvironment

**Figure 5:**
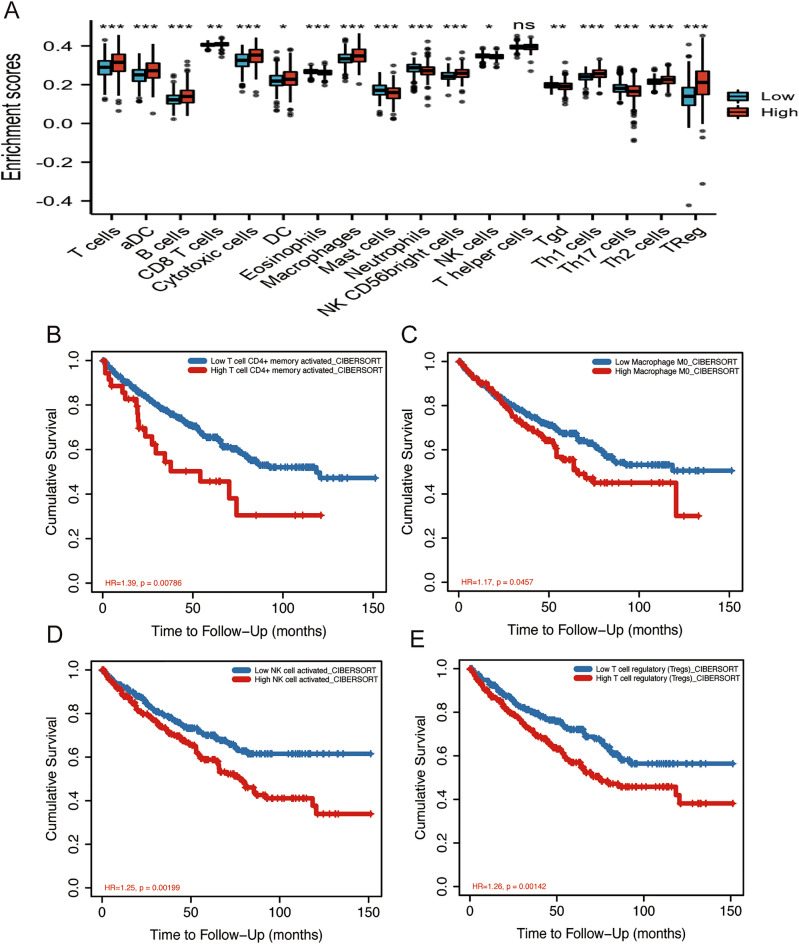
Analysis of immune cell infiltration. (**A**) The differences in immune cell infiltrations between low- and high-risk groups. The red and blue box represented the IRGPM high- and low-risk groups, respectively. The relationships between OS and (**B**) T cell CD4+ memory. (**C**) Macrophage. (**D**) NK cell and (**E**) T cell regulatory based on TIMER2.0.

To further reveal the role of IPGRM on the TIME, immune cells infiltrating in ccRCC patients were investigated via the GSVA algorithm. Among the immune cells, the high riskscore group significant positive associated with the proportional numbers of T cells, DC, Cytotoxic cells, B cells, aDC, Th1 cells, Th2 cells, Macrophages, CD8 T cells, Treg and natural killer (NK) CD56bright cells (Fig. 5A and Table S7). Meanwhile, we studied the prognostic value of selected important immune cells, with higher infiltration abundance of CD4+T cells (Fig. 5B), activated memory M0 Macrophage (Fig. 5C), and activated NK cell (Fig. 5D), and Tregs (Fig. 5E) was significantly negatively correlated with OS. In conclusion, IRGPM associated with the infiltration level of the majority of immune cells, showing that the IRGPM could potentially indicate the status of the TIME.

### The correlation between IRGPM and immunotherapy response

**Figure 6:**
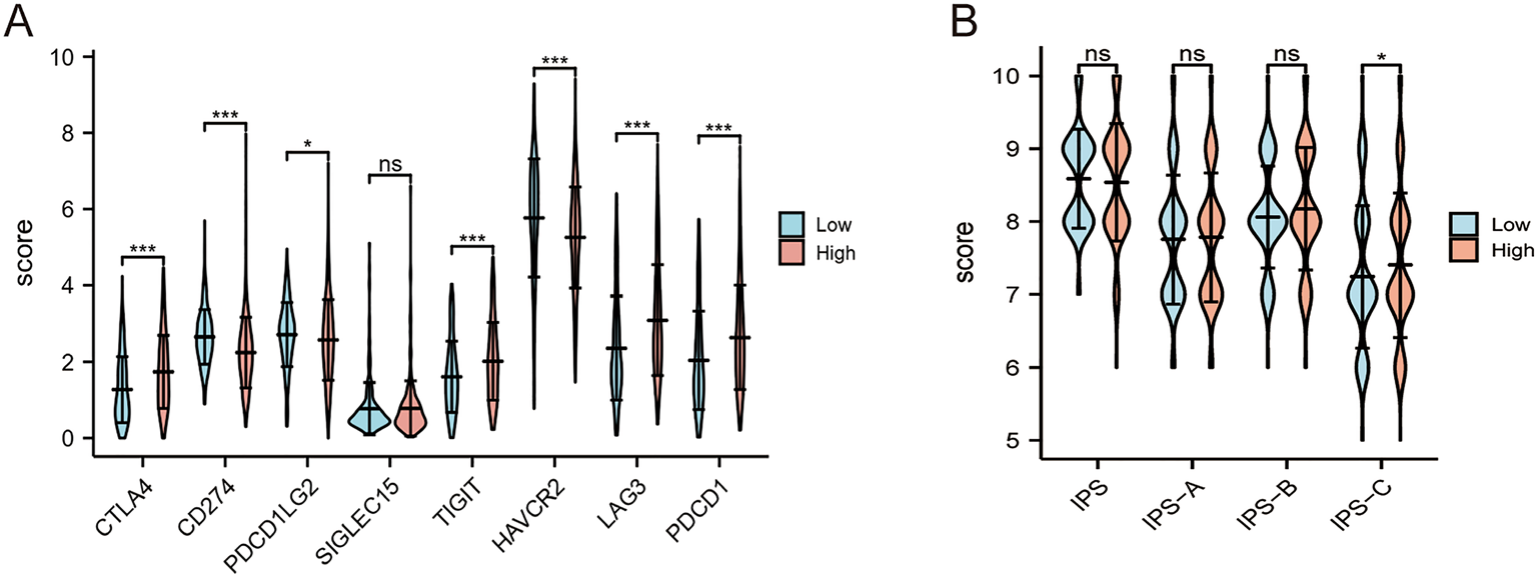
(**A**) The expression level of several salient checkpoints (CTLA4, CD274, PDCD1LG2, SIGLEC15, TIGIT, HAVCR2, LAG3, and PDCD1) between low- and high- riskscore groups based on TCGA-KIRC. (**B**) The difference in IPS between low- and high- riskscore groups. IPS: Immunophenoscore. IPS-A: CTLA4 blocker, IPS-B: PD1/PDL1/PDL2 blocker and IPS-C: CTLA4 and PD1/PDL1/PDL2 blocker.

Furthermore, the potential relationship between the IRGPM and immune checkpoints, including CTLA4, PD1/PDCD1, PDL1/CD274, PDCD1LG2, SIGLEC15, TIGIT, HAVCR2, LAG3, were investigated (Table S8). Figure 6A demonstrates that the low riskscore group was significantly positively correlated to CD274, PDCD1LG2, and HAVCR2, while it was negatively correlated to CTLA-4, TIGIT, LAG3, and PDCD1. The findings exhibited that the low riskscore group is involved in better immunotherapy efficacy of ICBs of CD274, PDCD1LG2, and HAVCR2, and their high expression is related to a better prognosis. The high riskscore group will benefit more from immunotherapy efficacy from ICBs of CTLA-4, TIGIT, LAG3, and PDCD1. IPS was found to be a promising index for evaluating the ICBs therapy response, and higher IPS indicated better immunotherapy response. A higher PD1/PDL1/PDL2 and IPS–CTLA4 blocker score was observed in the high riskscore group (Fig. 6B) The results of immunotherapy response are consistent with immunecheckpoint, a higher PDCD1 and CTLA-4 expression was observed in the high riskscore group.

## Discussion

In recent years, immunotherapy has gained popularity as a treatment for various cancers, including ccRCC^39–41^. Studies have reported that the immunological landscape of the ccRCC microenvironment could be a critical prognostic factor that should not be ignored to improve the potential for accurate treatment^18,19,42^. However, the efficacy of immunotherapy varies among individuals, and only a proportion of patients experience clinical benefits. Therefore, it is crucial to create a robust metric for predicting the survival of ccRCC patients and expand the repertoire of effective cancer immunotherapies.

In recent years, genomics and bioinformatics have made it possible to precisely determine molecular signatures. While some researchers have developed prognostic indicators based on miRNAs, lncRNAs, or mRNA, we believe that an immune gene-based signature is more appropriate for predicting the prognosis in immunotherapy^43,44^. Eleven optimal IRGs-based models were established as follows: *SAA1*, *IL4, PLAUR, PLXNB3, ANGPTL3, AMH, KLRC2, NR3C2, KL, CSF2,* and *SEMA3G.* Among these 11 IRGs, some have been found to be closely associated with immune cells. *SAA1* is involved in the activation of monocytes and macrophages during inflammation^45^. *IL-4* functions as a T-cell-derived B-cell growth factor^46^. *ANGPTL3* plays an active role in the Treg expansion function^47^. *KLRC4* participates in the antitumor immune role of the NK cells^48^. *NR3C2* and *CSF2* are correlated with macrophage polarization and activation^49,50^. Some genes might be implicated in immunomodulatory activities, such as *PLAUR*^51^*, PLXNB3*^52^, *AMH*^53^*, KL*^54^*, and SEMA3G*^55^*.* A previous study obtained data from the TCGA database and established a model based on 14 IRGs using the LASSO-COX method^18^. Unfortunately, the model suffers from platform bias and lacks validation data from other databases. Zhou et al. developed a prognostic model for immune-related genes pairs through bioinformatics analysis of papillary renal cell carcinoma; however, this model did not include clinical variables^19^.

To further assess the clinical effect of IRGPM, we studied its relationship with clinicopathological factors, IPS, and immune cell infiltration. First, we found that poorer pathologic state, higher histologic state, male sex, and higher TNM stage were associated with the high-risk group, suggesting that our prediction model is accurate in predicting the progression of ccRCC. Second, we assessed the proportion of immune cells in ccRCC samples. Studies have shown that the interaction between the microenvironment and the tumor is a determining factor in ccRCC progression. Therefore, we evaluated the potential of IRGPM to display the prognostic value of immune cells and immune cell infiltration. M0 macrophages and Tregs have been reported to promote tumor progression in various cancers ^56,57^, which are consistent with our study that high riskscore patients showed a higher enrichment of these cells. Interestingly, our study suggests that NK cells enhance the immune response and lead to a better prognosis based on TCGA-KIRC cohort (Fig. 5A). Conversely, analysis from TIMER in our study showed that high infiltration levels of activated NK cells is associated with poor prognosis (Fig. 5D). The potential reason of the inconsistent is that NK cells exert both oncogenic and tumor-suppressive effects in ccRCC. NK cells facilitate tumor growth and angiogenesis by releasing cytokines and growth factors in ccRCC. NK cells can directly recognize and killing ccRCC cells and activating other immune cells (T cells) to eliminate the ccRCC cells^58^. Another possible reason of the inconsistent is that the two results are based on different genomic data. Previously study demonstrated that enriched DCs were found to be closely associated with dysfunctional T cells, resulting in poor patient survival^59^. Similarly, our results indicated that the high riskscore group exhibited higher DC cell infiltration (Fig. 5A). Finally, we assessed immune checkpoint therapy in patients with ccRCC and found that the high riskscore group presented better IPS involving CTLA4 and PD1/PDL1/PDL2 combination blockades. This indicates that the high-risk group was more immunogenic to ICBs. This insight could guide the prediction of personalized cancer immunotherapy.

Yin et al. developed a gene signature to predict prognosis of ccRCC, while they only explore the response to anti-PD-1 therapy^60^. Zhang et al. constructed a gene-based model for prognosis prediction of ccRCC^61^. However, the study didn`t investigate relationship between the models and immune infiltration/immune checkpoints/ immunotherapy. Our study has several strengths compared with previously reported studies. First, the signature is confirmed and evaluated using multiple datasets, improving its reliability. Second, we performed several multifaceted studies that included discussions on the association of ICB and IRGPM with immune cells. Third, we established a nomogram for quantitative calculations, which is beneficial for clinical dissemination and application. Nonetheless, this study also had several limitations. First, our risk model was based on bioinformatics analyses of GEO and TCGA. To make this study more reliable, examining them experimentally in conjunction with clinical specimens is necessary. Furthermore, our study was retrospective in nature, and a prospective randomized trial should be designed to test and validate our hypotheses.

## Conclusions

In conclusion, we established an IRGPM for ccRCC to provide a better picture of the effect of immunotherapy and accurately predict the survival of patients with ccRCC. The IRG model provides clinicians with an effective tool for more precise treatment of patients with ccRCC.

### Supplementary Information

Supplementary Figures.

Supplementary Table S1.

Supplementary Table S2.

Supplementary Table S3.

Supplementary Table S4.

Supplementary Table S5.

Supplementary Table S6.

Supplementary Table S7.

Supplementary Table S8.

## Data Availability

The data that support the findings of this study are openly available found here: TCGA-KIRC (http://portal.gdc.cancer.gov/) and GEO-GSE29609 (https://www.ncbi. nlm.nih.gov/geo/).
