# Supplementary material for: A novel immune-related gene signature for predicting immunotherapy outcomes and survival in clear cell renal cell carcinoma
Source: Sci Rep. 2023 Nov 2;13:18922. doi: 10.1038/s41598-023-45966-8 (PMC10622518; doi:10.1038/s41598-023-45966-8)

Figure S1: Feature selection using LASSO logistic regression based on TCGA-KIRC cohort. (A) Lasso coefficient profiles of prognosis-related IRGs (B) Penalty plot of the LASSO mode, 10-fold cross validation based on minimum standards for the OS.


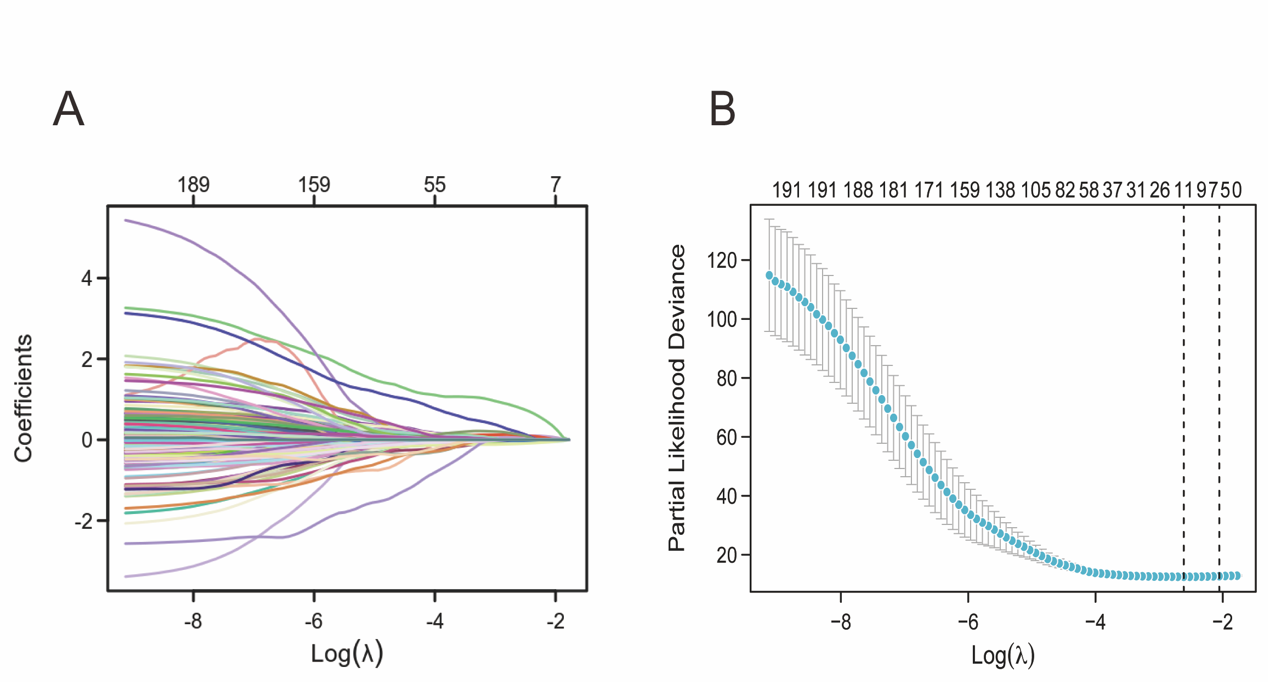


Figure S2. Forest plot showing multivariable Cox model of each gene based on TCGA-KIRC.

Figure S3. The Kaplan-Meier OS curves of low-risk group and high-risk group based on GSE29609.


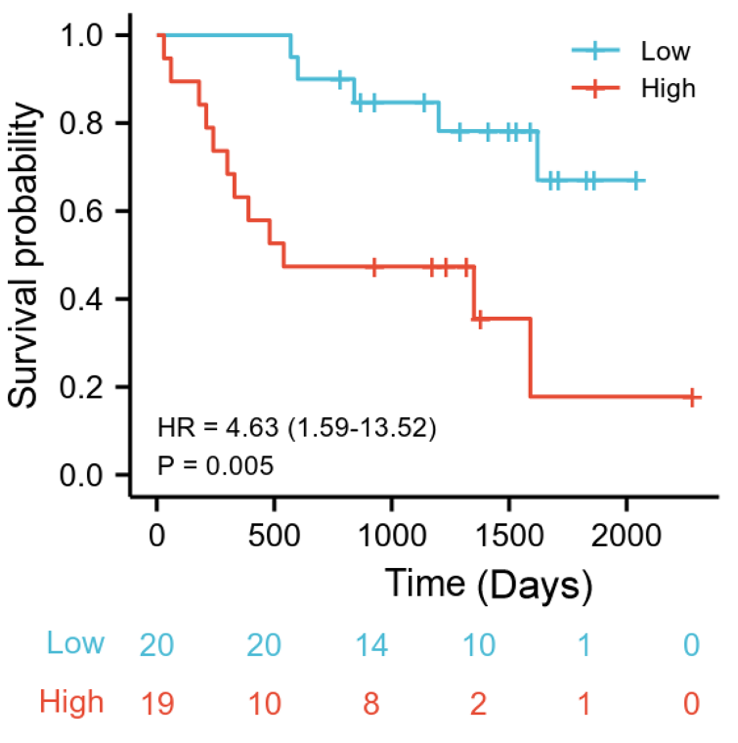

Supplement: Supplementary file 1 — Supplementary Figures. [file 41598_2023_45966_MOESM1_ESM.docx]
